# A new graphical method to display data sets representing biomechanical knee behaviour

**DOI:** 10.1186/s40634-015-0034-0

**Published:** 2015-08-28

**Authors:** Silvia Pianigiani, Jos Vander Sloten, Walter Pascale, Luc Labey, Bernardo Innocenti

**Affiliations:** IRCCS Istituto Ortopedico Galeazzi, via via R. Galeazzi 4, 20161 Milan, Italy; KU Leuven, Biomechanics Section, Celestijnenlaan 300 - bus 2419, Leuven, Belgium; KU Leuven, Mechanical Engineering Technology TC, Kleinhoefstraat 4, Geel, Belgium; BEAMS Department, Université Libre de Bruxelles, Av. F. Roosevelt, 50 CP165/56, Bruxelles, Belgium

**Keywords:** Graphical method, Biomechanical analysis, Knee

## Abstract

**Background:**

When researchers describe data from their studies, there is no rule defining the best way to represent results. Therefore, collecting and explaining results from personal research or understanding data from publications is not always straightforward. These issues are even worse in fields such as biomedical engineering, where researchers from different backgrounds, usually engineers and surgeons, need to interact and exchange information. For these reasons, the purpose of this study is to introduce and illustrate an innovative method to represent, concisely and intuitively, biomechanical knee behavior, called KneePrints.

**Methods:**

To test the KneePrints method, a huge amount of data from previously published sensitivity analyses were used and represented both with conventional techniques and with this new graphical method. Then, a survey has been distributed among different international specialists in the orthopedic field, such as surgeons and researchers. In the survey, interviewees were asked to select the favorite method that addressed to be the most effective to show the same results.

**Results:**

Collecting the outcomes from the survey, the KneePrints method resulted to be more effective than standard graphs, such as tables and histograms. KneePrints method has been selected to be clearer in representing outputs and more immediate in results understanding independently from the occupation of the interviewees by the survey. The general preference for the KneePrints is 63 %, up to 74 % being surgeons’ choice.

**Conclusions:**

The innovative KneePrints method has been endorsed to be effective in representing and making more understandable knee joint outputs. This method can be extended also to other topics.

## Background

When researchers describe data from their study, there is no accepted rule that defines the best way to represent results. Therefore, collecting and explaining results from personal study, or understanding data from the literature, is not always straightforward. These issues are even worse in the biomedical engineering field in which people with different backgrounds, usually engineers and clinicians, need to interact and exchange information.

By definition, graphical displays complement verbal discourse in written documents and in oral presentations as a more powerful form of effective redundancy. Through spatial relationships and potential richness of detail, they provide insights in ways that text cannot hope to match (Doumont 2009).

Graphs are mainly used to show comparisons between data sets, overlapping of data, compositions of data, correlations among variables, or evolutions of a variable (Best 2005).

The graph is a visually reproduced concept that is aimed to bridge the gap between meaning across languages and different research fields.

Designing good charts presents more challenges than tabular display as it draws also on the artistic talent of the scientist; in fact, the complete knowledge and understanding of own data is necessary together with a good sense of how the audience will visualize and understand the chart’s graphical elements independently from the structure of the dataset as, often, the structure of the data set largely suggests the type of graph to be selected (Coles 1997, Few 2004). Poor structure ruins otherwise effective graphs by accidentally distorting the data, making them hard to read, or distracting the readers if purposefully misleading (Doumont 2009).

Unfortunately, it could happen that researchers have a straightforward overview of their represented results while the reader can not immediately understand the meaning of the results section of these papers, especially when there are mainly graphs and images to show the outputs.

As there is no standard method to be used to show data, authors can decide which is the best method for presenting their work, but the chosen method may not be fully understandable for the readers.

Sometimes, a compromise between details and accuracy is made in showing results, especially when a big amount of data has been collected.

In literature, papers usually present data through tables, pie charts, and histograms to show all the results, but often confuse the reader or overflow him with information (Innocenti et al. 2011, Pianigiani et al. 2012).

Looking at literature about biomechanics of knee joint, a large number of studies has been published. These studies are based on different techniques such as experimental tests and in vitro tests (Arnout et al. 2014, Delport et al. 2013, Heesterbeek et al. 2014), in vivo measurements (Battaglia et al. 2014, Fregly et al. 2012, Kutzner et al. 2011), numerical analyses (Fitzpatrick and Rullkoetter 2014, Innocenti et al. 2014, Zelle et al. 2011), imaging or biologic tests (Victor et al. 2009, Worsley et al. 2011, Zdero et al. 2001). In these works, knee movements and forces are expressed in function of several motor tasks that can be performed both during daily activities and in some extreme situations, such as sport activities, and comparing healthy or pre-operative knee conditions with post-operative configurations. The method of representation is often typical for that specific area in which the analysis has been performed, but less common in other fields and sometimes also dependent on the technique in use.

Aiming to find an innovative graphical method to show datasets representing knee biomechanics, a new technique called “KneePrints” has been proposed. Thanks to its customizable nature, the KneePrints graph can be adopted for several situations and also to represent data from sensitivity analyses.

Introducing the KneePrints, the first aim is to define a new methodology to bridge the gap between making presentable and explainable data, about knee biomechanics, for the writer and so, making the readers able to manage the full flow of represented biomechanics data and to improve its comprehension.

To demonstrate the efficacy of this new method, it has been tested on an already published set of data (Innocenti et al. 2011). To evaluate the perception of different audiences in the biomedical field, a survey was proposed to surgeons and researches. In the survey, this new technique has been compared with more conventional presentation methods for the same data set. In the survey, distributed by hand and on line to international operators in the biomechanical field, a detailed description of the proposed method was also provided.

## Methods

The KneePrints graph aims to represent how the joint’s biomechanics changes according to daily motor tasks inputs or any other variations, such as, but not limited to, changes in anatomical factors or knee implant component types and positions.

**Fig. 1:**
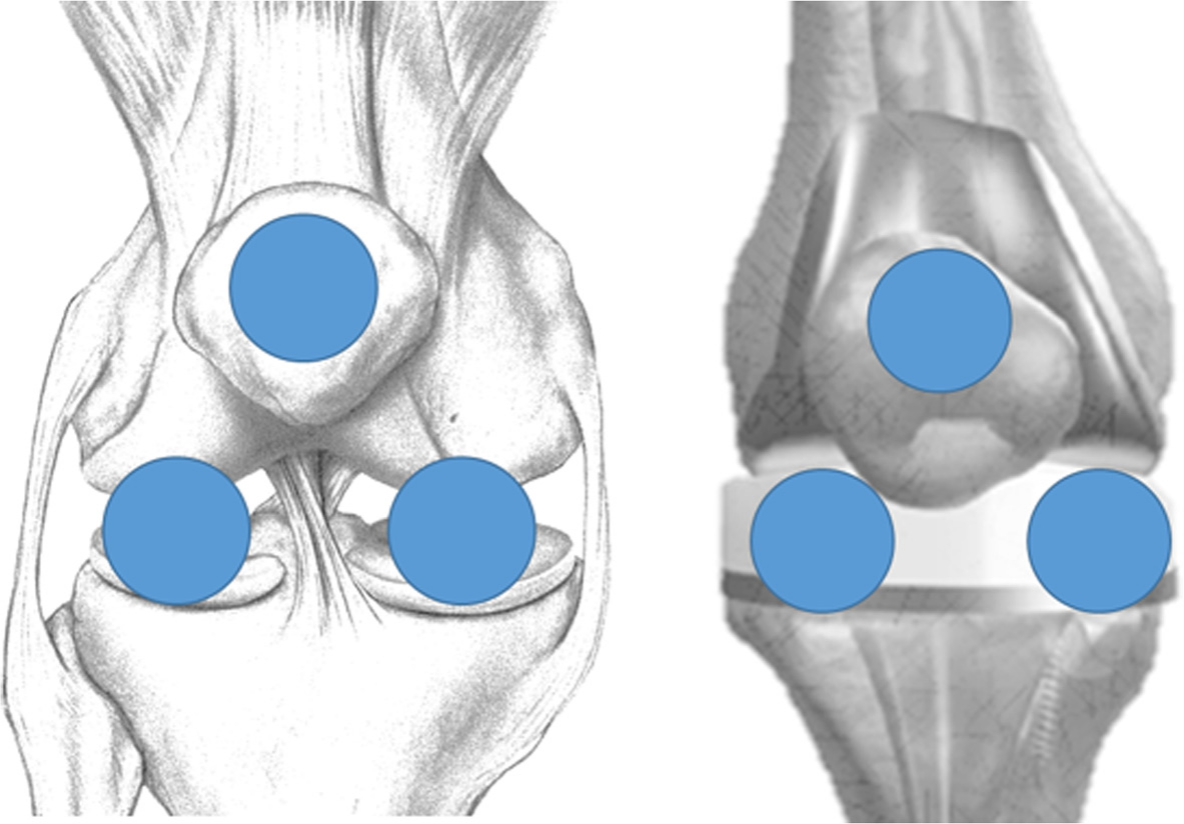
On the selected background, the position of the circle graphs indicates directly which response is described, for example, the medial tibio-femoral contact force graphic is placed on the medial interface between the femur and the tibia bones for the native joints and femoral and tibial component for a knee with a total knee arthroplasty

As shown in the template in Fig. 1, the outputs are directly represented in a circle positioned over the analyzed structures located in the background both for a native knee and for a knee with a total knee arthroplasty.

The KneePrints can be made after a conventional table is filled with all the outputs recorded from standard or sensitivity analyses. Once all the calculations are terminated, in case of standard analysis, a relative coloured bar can be defined to take into account the range and the values of the outputs. The coloured bar can indicate, for example, with a green colour the reference configuration, and with a red and a blue colour respectively an increase or a decrease of the output value with respect to the reference value.

**Fig. 2:**
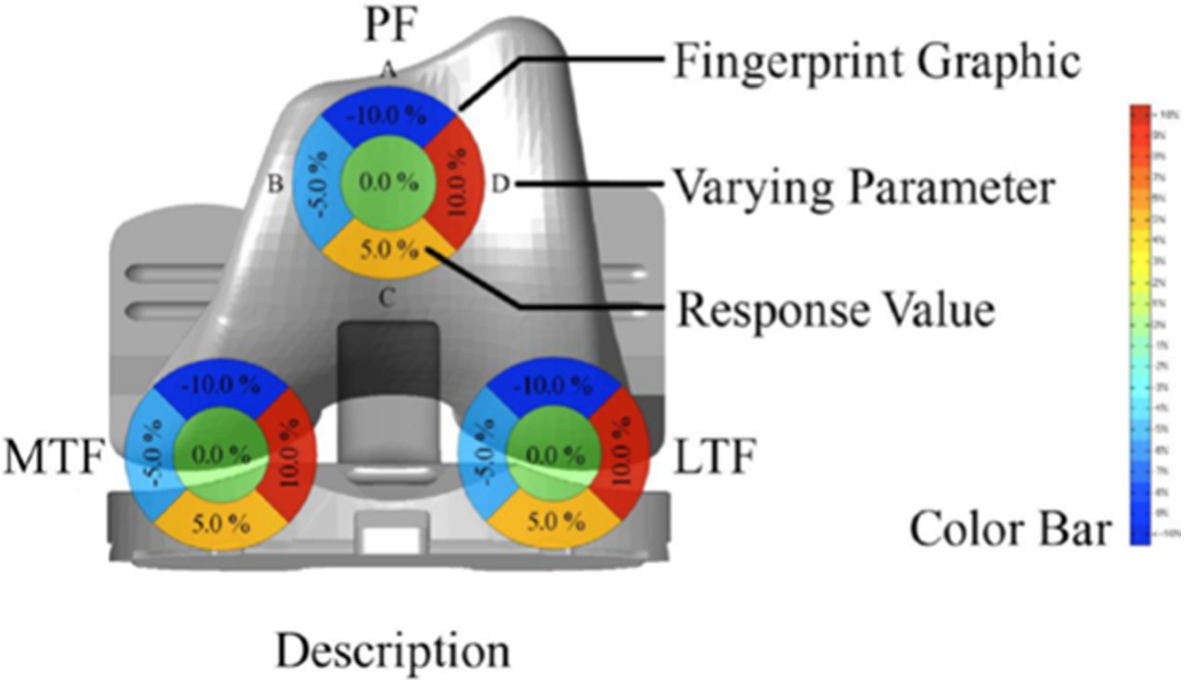
The standard graphic proposed in this study consists of circular graphics overlaid to a specific background. For example, for the KneePrints after a sensitivity analysis, circular graphs are sited on a specific total knee arthroplasty design at the patellofemoral (PF), medial tibiofemoral (MTF) and lateral tibiofemoral (LTF) locations. The description may indicate a fixed parameter for combined configurations studies or the varying parameter for single configurations. A, B, C, and D indicate the varying parameter configuration and the response value is displayed in text and in colour (according to the colour bar)

In the case of sensitivity analyses, a comparison among each deviated configuration and the reference can be performed and the result can eventually be represented in percentage of variation. While filling the KneePrints graph is quite straightforward for standard analysis, for a multi-parametric analysis more details should be added to improve the findings understanding. For example, the circle graphs could consist of two (or more) concentric circles indicating, for example, the inner circle the reference configuration, while the outer rings can be subdivided to account for multiple simulated configurations considered. An example is shown in Fig. 2. For the sake of clarity, in each section of the circular graph, the value of the outputs or its change expressed in percentage should be optionally indicated. Then, according to the previously defined coloured bar, that indicates different thresholds of changes, the inner part of the graph is green filled, while each section of the outer ring is coloured to indicate the relative change of the output. Moreover, a label describing which altered configuration is considered in each part of the ring could be added (Fig. 2).

**Fig. 3:**
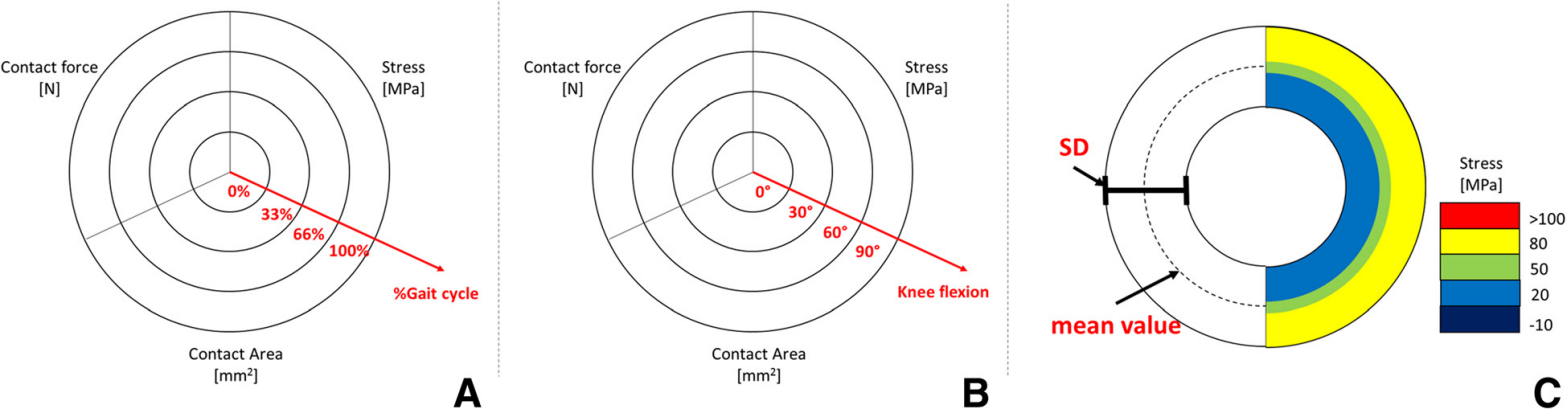
Possible solutions to express different outcomes in function of the percentage of a simulated task, i.e. gait cycle (**a**), or a certain knee flexion (**b**). Data can be expressed as absolute values (**a**,**b**) or expressed as mean values and their standard deviations (**c**). **c** shows an example of application for stress outputs for which, i.e., mean value is 50 and SD =30

The previous description is mainly dedicated to sensitivity analysis, however the KneePrints can also be adopted to express data from other analysis such as in-vivo or in-vitro tests. In these situations it is often important to provide an overview of one or several outputs with respect to the knee flexion achieved during a specific task. For these cases, Fig. 3a and 3b show how the ring to be imposed on the back ground could be subdivided. In detail, both figures are subdivided in three main regions that are dedicate to different outputs. Each region is then subdivided in portions of rings that are referred to different percentage of the analyzed motion, i.e. Fig. 3a shows a possible subdivision for the gait cycle, or different knee flexions (Fig. 3b) achieved during the simulated task. On the other hand, often it is important to express mean values outputs and their standard deviations. Figure 3c show a possible template for such data representation.

To generate a KneePrints graph, the user can decide to use the favorite graphical software. The method is not strictly linked to a particular software.

Once the KneePrints is obtained, there is a large amount of information to could be extracted. First of all, looking at the picture, the reader will see if the analysis is about a native knee or a knee with a prosthesis. In the case of a knee with a prosthesis, the reader can visualize its design and its features. Second, in the case of a standard analysis, data concerning knee contact forces or knee kinematics are described in the areas relative to tibio-femoral and patello-femoral responses, or also ligaments outputs, if it is needed. Third, if outputs from a sensitivity analysis are shown, the reader can obtain additional multiple information: reading only the green circle he can understand the standard behavior of the joint, while by observing the outer ring he can understand how and how much a varying parameter can affect the analyzed output. The use of such coloured bar will help the reader in understanding more intuitively which is the most affecting parameter and in which area of the joint.

To prove the efficacy of the KneePrints method, the characteristic responses of the maximum patello-femoral and medial and lateral tibio-femoral contact forces after a sensitivity analysis, as reported in Innocenti et al. (2011), have been considered. In this published paper, conventional representation methods, such as tables and histograms, have been used to represent a huge amount of data after 96 analyzed configurations.

In order to detect if the proposed method is valid and more understandable for the readers than standard graphical methods, a survey among people in the biomedical field was conducted. Face to face interviews took place during international conferences and trough an on-line survey (accessible by www.surveymonkey.com/s/PM6SYNL). The interviewed people were not conditioned during the survey and their results are anonymous.

People were asked to answer three questions after being provided with an explanation about KneePrints.

The questions were:

- The gender (male, female, prefer to not reply);
- The occupation (Medical doctor, Engineer/Researcher, Other);
- The preference among Tables, Histograms or KneePrints to represent the same outputs from a sensitivity analysis (Innocenti et al. 2011).

**Fig. 4:**
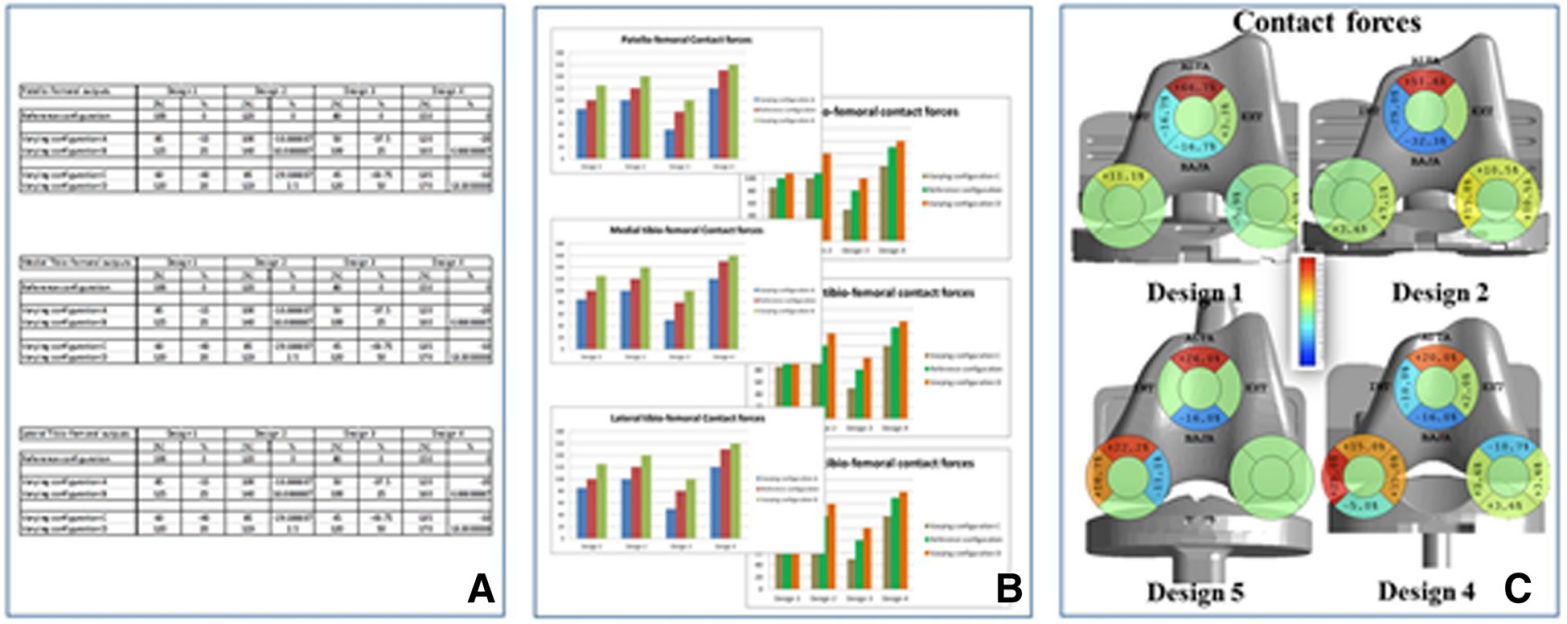
Possible graphical methods to represent the outputs after the same sensitivity analysis: **a** table, **b** histogram, **c** KneePrints

Figure 4 shows how the final question of the survey that has been proposed to compare three different graphical methods in expressing the same outputs.

Replies from the survey were collected and subdivided by gender and occupation of the interviewees. In order to calculate the probability distribution of the outcomes, a multinomial distribution statistical analysis has been performed determining, for all the groups, singularly and overall an interval of confidence at 95 %.

## Results and discussion

A total of 130 people replied to the survey. 80 % were male and 17 % were female (3 % preferred to not indicate his/her gender), 30 % were medical doctors, 63 % were engineers or scientists, 6 % were other kind of workers in the biomedical field and 1 % preferred not to answer.

**Fig. 5:**
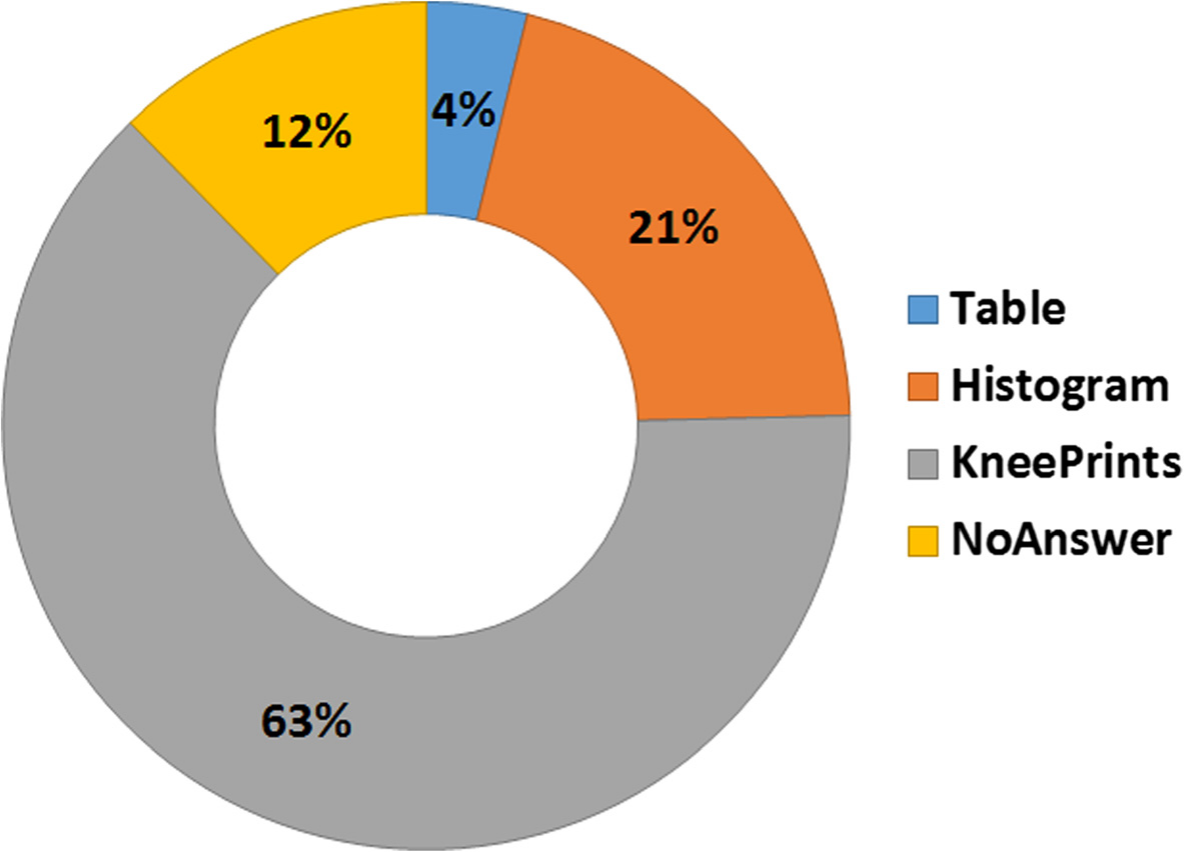
Results from the survey about the preferred method, among table, histogram or Kneeprints, for representing data outputs after the same sensitivity analysis

**Fig. 6:**
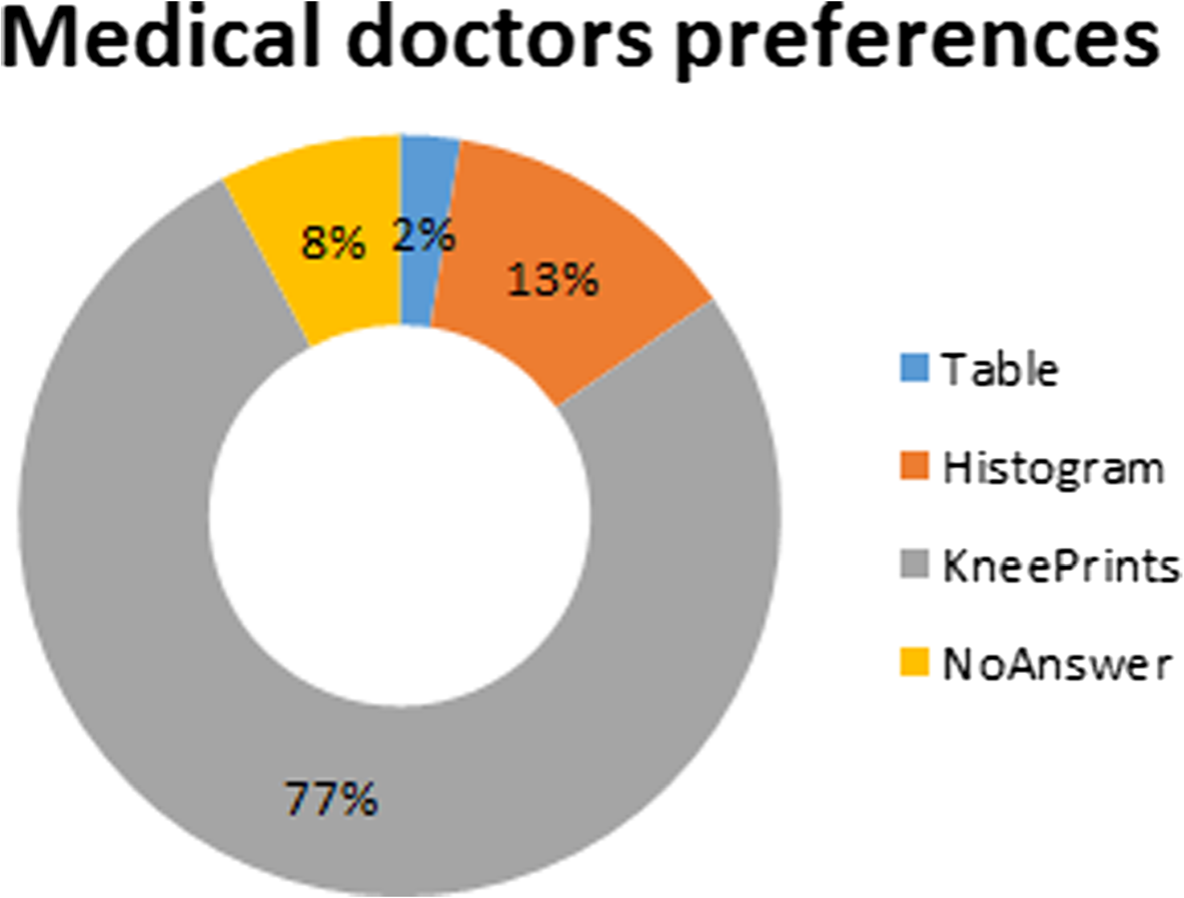
Results from the survey about the preferred method of representation by medical doctors

**Fig. 7:**
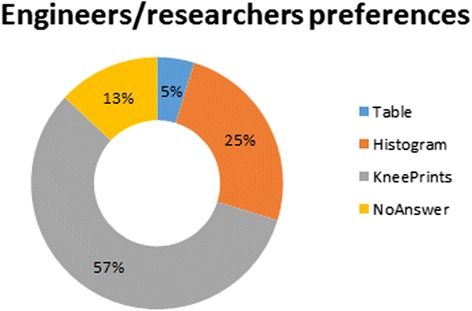
Results from the survey about the preferred method of representation by engineers/researchers

**Fig. 8:**
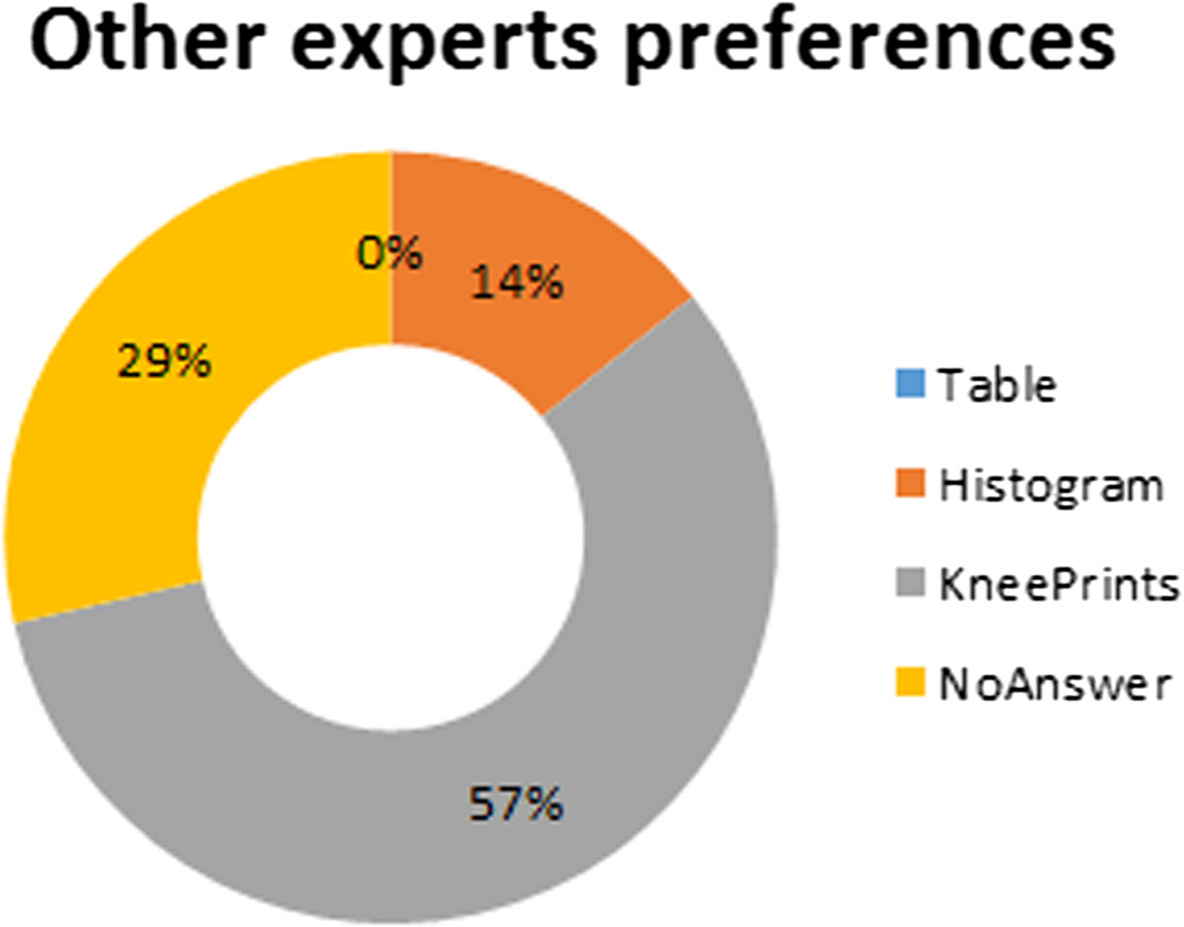
Results from the survey about the preferred method of representation by other expert in the biomedical field

Figures 5 shows that the KneePrints method is the favourite among the proposed data presentation formats in the survey with a preference of 63 %. The Kneeprints is the favourite independently from the type of interviewed operator: 77 % of the medical doctors (Fig. 6), 57 % of the engineers/researchers (Fig. 7) and 57 % of the other group (Fig. 8) preferred the KneePrints to the other two graphical methods.

After performing a multinomial distribution, based on the 95 % confidence interval, KneePrints is expected to be selected with a probability of 62–77 % for the overall group; in particular surgeons are expected to choose it with a probability of 75–97 %.

As parameter variation studies for knee biomechanics understanding become more common, standardized graphical representations of the results will become necessary for the comparison and the validation of different analyses.

In this paper, the concept of KneePrints has been introduced as a tool to characterize and graphically convey outputs, also from a sensitivity analysis, describing possible knee biomechanics alterations related to patient anatomical factors and/or surgical variability in implant component position and orientation of a possible implanted total knee arthroplasty design.

The challenge of a method to represent results intuitively is dual because it must suit the writers’ needs and the readers’ perception. In particular, readers’ perception can be affected by different backgrounds, such as in the biomedical field where both medical doctors and engineers are mutually involved. A graphical approach which all at once prints the responses of a specific case to same factors variability is strongly suggested.

To show how the standard KneePrints developed graphic should be used to report future numerical sensitivity studies, the maximum patello-femoral and tibiofemoral contact force, after commonly reported surgical variations in total knee arthroplasty components alignment obtained in a recent study (Innocenti et al. 2011), were presented as application example.

According to the results, related to the proposed survey, the KneePrints method is generally the preferred, with respect to the standard used graph methods, from 7 on 10 interviewed people. Hence, KneePrints covers the aim to be concise and intuitive for different kinds of operators in the biomedical field.

The new proposed graphical method has been tested in the survey reporting only one case of already published data for a sensitivity analysis. However, testing it with different data sets could help in understanding its efficacy also for other typologies of outcomes, i.e. experimental outcomes describing mean values and standard deviations.

On the other hand, from the audience comments, the KneePrints resulted more effective, especially for the way results are directly reported in the region of interest, without many labels or captions, and for the use of intuitive colors that immediately highlight the worst scenarios, so can be hypnotized that similar preference can be achieved also representing other datasets. In particular, the feedbacks from the interviewed clinicians were the most favorable expressing that finally also data from studies leaded by engineers were immediately and clearly understandable and could be used as a guideline to improve the clinical research.

In this paper an application based on a numerical analysis was introduced, but the method can be also used for other kinds of analysis, such as experimental tests and gait analysis for the knee. For the sake of clarity, the authors would like to suggest the use of this new method also in representing already published data from different papers in order to employ a unique graphic method to compare them.

Moreover, the KneePrints is a particular application of a more general graphical method. The suggestion of its application for other joints biomechanics understanding or other research fields is thus foreseen. When the same approach is needed for other fields, the same indications provided to obtain the KneePrints should be followed changing some features accordingly to the content, such as, for example, the background of the graph or the labels of the different rings.

Depending on the demand of using the new proposed method, future developments of this study, that mainly provides guidelines on how obtaining this representation of data, should include the generation of an ad-hoc software that could be helpful to quickly generate this sort of graphs and let them even more standardized.

## Conclusions

The purpose of this study was to introduce and illustrate an innovative method to represent, concisely and intuitively, biomechanical knee behavior, called KneePrints. KneePrints has been endorsed to be more efficient than standard graphical methods. For these reasons, the KneePrints method can be proposed to bridge the gap between research outputs and applications in the orthopedic field.
